# N-Doped TiO_2_-Coated Ceramic Membrane for Carbamazepine Degradation in Different Water Qualities

**DOI:** 10.3390/nano7080206

**Published:** 2017-07-31

**Authors:** Enbal Luster, Dror Avisar, Inna Horovitz, Luca Lozzi, Mark A. Baker, Rossana Grilli, Hadas Mamane

**Affiliations:** 1 The Water Research Center, School of Earth Sciences, Faculty of Exact Sciences, Tel Aviv University, Tel Aviv 69978, Israel; lustere@gmail.com (E.L.); droravi@post.tau.ac.il (D.A.); HorovitzInna@gmail.com (I.H.); 2 Department of Physical and Chemical Sciences, University of L’Aquila, Via Vetoio, I-67100 L’Aquila, Italy; luca.lozzi@aquila.infn.it; 3 The Surface Analysis Laboratory, Faculty of Engineering and Physical Sciences, University of Surrey, Guildford, Surrey GU2 7XH, UK; m.baker@surrey.ac.uk (M.A.B.); rossana.grilli@blue-scientific.com (R.G.); 4 School of Mechanical Engineering, Faculty of Engineering, Tel Aviv University, Tel Aviv 69978, Israel

**Keywords:** photocatalytic membrane, N-doped TiO_2_, water treatment, water quality, radical scavenging, membrane regeneration

## Abstract

The photocatalytic degradation of the model pollutant carbamazepine (CBZ) was investigated under simulated solar irradiation with an N-doped TiO_2_-coated Al_2_O_3_ photocatalytic membrane, using different water types. The photocatalytic membrane combines photocatalysis and membrane filtration in a single step. The impact of each individual constituent such as acidity, alkalinity, dissolved organic matter (DOM), divalent cations (Mg^2+^ and Ca^2+^), and Cl^−^ on the degradation of CBZ was examined. CBZ in water was efficiently degraded by an N-doped TiO_2_-coated Al_2_O_3_ membrane. However, elements added to the water, which simulate the constituents of natural water, had an impact on the CBZ degradation. Water alkalinity inhibited CBZ degradation mostly due to increase in pH while radical scavenging by carbonate was more dominant at higher values (>200 mg/L as CaCO_3_). A negative effect of Ca^2+^ addition on photocatalytic degradation was found only in combination with phosphate buffer, probably caused by deposition of CaHPO_4_ or CaHPO_4_·2H_2_O on the catalyst surface. The presence of Cl^−^ and Mg^2+^ ions had no effect on CBZ degradation. DOM significantly inhibited CBZ degradation for all tested background organic compounds. The photocatalytic activity of N-doped TiO_2_-coated Al_2_O_3_ membranes gradually decreased after continuous use; however, it was successfully regenerated by 0.1% HCl chemical cleaning. Nevertheless, dissolution of metals like Al and Ti should be monitored following acid cleaning.

## 1. Introduction

Public health concerns, increased worldwide environmental awareness and improved analytical and technological capabilities are the main driving forces in improving water quality. The use of solar irradiation to directly convert photons into photochemical energy is considered a sustainable approach to deliver water disinfection efficiently and economically [1]. The semiconductors activated by sunlight irradiation (i.e., photocatalysis), present an attractive technology for disinfection and pollutant degradation in water [2,3]. Photocatalysis can be implemented for numerous applications and products as self-cleaning surfaces, systems for treatment of air and water, sterilization, and hydrogen production [4].

Titanium dioxide (TiO_2_) is one of the most commonly studied nano photocatalysts for removing/breaking-down contaminants (e.g., pharmaceuticals, pesticides, antibiotics, endocrine-disrupting compounds) in water [3]. The TiO_2_ based advanced oxidation process (AOP), is used for oxidation of a broad range of contaminants by the very reactive, short-lived and non-selective hydroxyl radicals. Among the various configurations for photocatalytic reactors (e.g., packed bed, fluidized bed, falling film, nanotube Array-Based Reactor), photocatalytic membrane reactors (PMR) can benefit from combined membrane filtration and photocatalysis in a single step. In the thin-film PMR hybrid configuration (as opposed to PMR with catalyst suspended), the catalyst is coated on or within the membrane pore matrix, and stimulated by direct exposure to light [5,6]. When designing a photocatalytic reactor for water treatment, water composition must be considered among the operational parameters to ensure treatment efficiency and corresponding optimization. The chemical composition of different water types can vary significantly and therefore it is crucial to understand the impact of the dominant species on the photocatalytic process. The presence of anions (e.g., bicarbonate and chloride), cations (e.g., calcium, magnesium), and natural organic matter (NOM) can enhance or suppress the photocatalytic efficiency through mechanisms such as competition for surface adsorption or for hydroxyl radicals and light screening by organic substances [2]. Conflicting results have been reported by various researchers on the effect of ions and NOM on the photocatalytic activity, mainly due to the differences in experimental system, experimental design, and conditions [7,8]. For example, carbonate/bicarbonate anions react with the hydroxyl radical to transform to species that are selective and thus less desirable than the corresponding anion radicals. However, at high concentrations these radicals may also prove significant in the photocatalytic reaction due to their stability and longer lifetime [9]. Another route to inhibit the photocatalytic process is the adsorption of ions at the active sites, which depends on the species concentration as well as type, solution pH, and the isoelectric point (IEP) of the catalyst. Natural organic matter (NOM) in water can enhance the photocatalytic reaction through its photosensitization properties [10] or inhibit by scavenging of surface generated hydroxyl radicals [11].

This study uses α-Al_2_O_3_ microfiltration (MF) membranes as a substrate for highly efficient N-doped TiO_2_ coating activated by UV-visible light. The characterization and impact of physical parameters as pore size, wavelengths, flow-rate, and permeability of the N-doped TiO_2_-coated membrane reactor have been reported by Horovitz et al. [12]. Membrane porous structure enabled improved mass transfer of reactants to the catalytic surface as a consequence of in-pore convection and enhanced diffusion. The modification of the nanomaterial by nitrogen facilitated more efficient utilization of solar irradiation. From a practical standpoint of process operation of fixed-film PMR on-site, it is vital to consider factors like water quality, membrane regeneration, and membrane fouling.

The goal of this study was to demonstrate the impact of typical parameters in water quality on the degradation efficacy of the organic micro-pollutant model compound, carbamazepine (CBZ), using N-doped TiO_2_ coated MF membrane. The effect of water quality parameters on the photocatalytic activity (PCA) were examined by discrete addition of selected dissolved species present in the water at various concentrations. Moreover, for practical applications, regeneration of the photocatalytic membrane was investigated by subjecting the membranes to standard membrane chemical cleaning after continuous use.

## 2. Results and Discussion

### 2.1. Effect of Water pH

**Figure 1:**
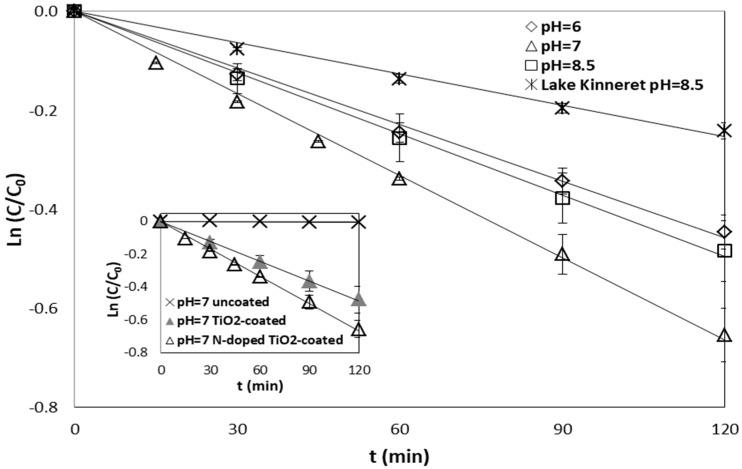
Carbamazepine (CBZ) degradation (C_0_ = 1 mg/L) as a function of pH (6, 7, and 8.5) in buffered deionized (DI) water and in Lake Kinneret water (pH 8.5) by N-doped TiO_2_-coated Al_2_O_3_ membrane under irradiation (The inset depicts: CBZ degradation by uncoated Al_2_O_3_, TiO_2_-coated Al_2_O_3_ and N-doped TiO_2_-coated Al_2_O_3_ membranes).

Figure 1 illustrates the efficiency of N-doped TiO_2_-coated Al_2_O_3_ membranes towards degradation of 1 mg/L CBZ, at various pH ranges (6–7, and 8.5) relevant to natural water and in natural surface water sampled from Lake Kinneret (Sea of Galilee, Israel). The water pH was adjusted by 1 mM phosphate buffer saline (PBS) to obtain a pH range between 6 and 7, and by 2 mM borate buffer saline (BBS) to obtain a pH value of 8.5. The pH of Lake Kinneret water was measured to be 8.5 and the most significant measured water quality parameters were: alkalinity (84 mg/L as CaCO_3_), hardness (280 mg/L as CaCO_3_), chlorides (310 mg/L Cl^−^), and dissolved organic carbon (DOC, 4 mg/L). Photocatalytic activity was in the following order: pH = 7 > pH = 8.5 = pH = 6 > Lake Kinneret. CBZ degradation was significantly inhibited in Lake Kinneret water as opposed to buffered deionized water (DI) water at the same pH (8.5), with ~44% decrease in reaction rate. These results were expected according to the inhibiting effect found in the literature for water containing alkalinity, NOM, and other species [13]. Several water dissolved species are examined for their impact on the photocatalytic process in the following sections. The differences in CBZ removal at pH 6 and 8.5 are not statistically significant (*p* > 0.05).

The ionization state of the photocatalytic surface (i.e., N-doped TiO_2_ film) is protonated under acidic conditions and deprotonated under alkaline conditions. The isoelectric point (IEP), where the total negative and positive charge on the surface is zero, of TiO_2_ nano-particles (Degussa P25) is at pH ~ 6.3 [14] while the IEP of pure α-Al_2_O_3_ is at 8.75–9.1 [15]. Nevertheless, N-doped TiO_2_ surface coverage on Al_2_O_3_ membrane was reported in our previous study to be ~80%, therefore the TiO_2_ coating determines the surface charge [12]. Zhou et al. reported IEPs of 6.1 and 4.0 for α-Al_2_O_3_ and TiO_2_-modified α-Al_2_O_3_ membranes, respectively [16]. Similar values were found by Zhang et al. for Al_2_O_3_-TiO_2_ composite membranes, with IEPs ranging from 6.1 to 4.1, with increase in TiO_2_ content. Therefore, it can be assumed that N-doped TiO_2_-coated Al_2_O_3_ will be mostly negatively charged at pH > 6 [17].

Water pH can affect the electrostatic interaction between the catalyst and organic molecules. For instance, TiO_2_-mediated photocatalytic degradation of 2-chlorophenol decreased with increasing pH values [18,19]. At pH below the IEP, chlorophenols and their transformation products are generally negatively and neutrally charged, while the surface of TiO_2_ is net positively charged, and can result in adsorption of chlorophenols and enhance photocatalytic degradation. However, in other studies, chlorophenols were insignificantly adsorbed onto TiO_2_ due to competition with water for adsorption sites and therefore, alkaline conditions improved photocatalytic efficiency for degradation of chlorophenols [20,21].

At low pH values, the positive holes are the main oxidation species, and at neutral or high pH hydroxyl radicals (OH·) are the dominant species. Consequently, as the pH level rises more available hydroxyl ions on the catalyst surface result in formation of additional OH· [3,22,23,24]. Avisar et al. found that the PCA of CBZ was enhanced at high pH using N-doped TiO_2_ photo-catalytic thin films on glass surfaces [2]. Conversely, Achilleos et al. found that TiO_2_ photocatalytic degradation of CBZ was the highest at ambient pH (~5.9) and reduced at acidic or alkaline conditions (pH 3–10) with a more pronounced reduction under alkaline conditions [25]. Vogna et al., however, showed that the degradation of CBZ, under UV/H_2_O_2_, was not affected by varying the pH solution between values ranging from of 2 to 8 [26]. As CBZ is neutral at a wide pH range (see Table 5), the generation of oxidative species on the photocatalytic surface is probably a result of pH change via surface charge. The difficulties in interpretation of the CBZ rate constant at various pH values may be attributed to complexity in quantifying the zeta potential for coated porous membrane and in determining the interactions between the substrate (α-Al_2_O_3_ membrane coated with N-doped TiO_2_) and CBZ. Nevertheless, even though the porous membrane surface is negatively charged while CBZ is neutral, slightly alkaline or acidic pH values reduced the rate constant in comparison to neutral pH.

Control experiments showed negligible effect of irradiation on CBZ degradation (i.e., using uncoated Al_2_O_3_ membranes) after 120 min exposure (<1%, see inset in Figure 1). In addition, N-doped TiO_2_-coated Al_2_O_3_ membrane was found to be superior to TiO_2_-coated Al_2_O_3_ membrane towards CBZ photocatalytic degradation. Detailed discussion on the advantage of N-doped TiO_2_-coated Al_2_O_3_ compared to unmodified TiO_2_-coated Al_2_O_3_ can be found in our previous work by Horovitz et al. [12].

### 2.2. Water Hardness

**Figure 2:**
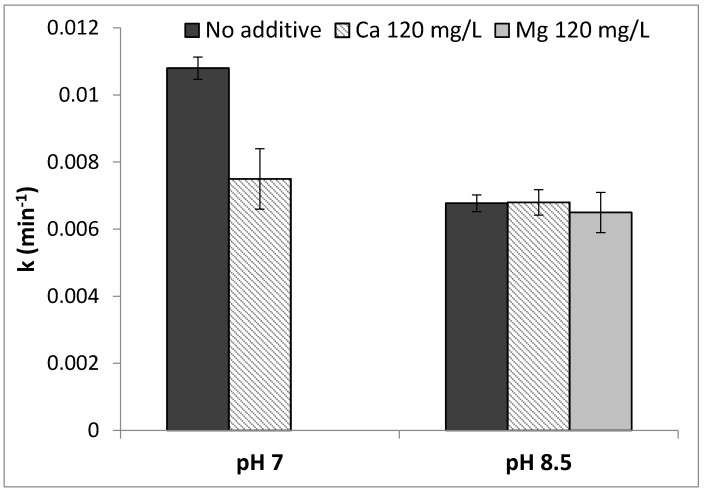
CBZ degradation by N-doped TiO_2_-coated Al_2_O_3_ membranes, without (no additive) and with addition of 120 mg/L Ca^2+^ (pH 7 and 8.5) and 120 mg/L Mg^2+^ (pH 8.5).

The effect of water hardness (i.e., addition of Ca^2+^ and Mg^2+^ in solution) on PCA of N-doped TiO_2_-coated Al_2_O_3_ membrane is presented in Figure 2 and was examined under the following conditions:

- Addition of 120 mg/L Ca^2+^ to DI water spiked by 1 mg/L CBZ and pH = 7 adjusted by 1 mM PBS.
- Addition of 120 mg/L Ca^2+^ to DI water spiked by 1 mg/L CBZ and pH = 8.5 adjusted by 2 mM BBS. Phosphate buffer could not be used in this case due to the precipitation of CaPO_4_.
- Addition of 120 mg/L Mg^2+^ to DI water spiked by 1 mg/L CBZ and pH = 8.5 adjusted by 2 mM BBS. Borate buffer was used to create identical conditions to those in the experiment with calcium.

**Figure 3:**
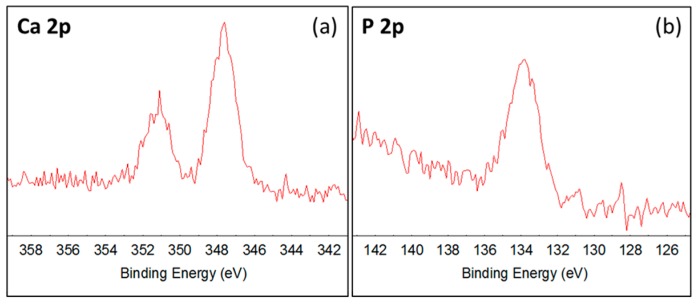
XPS core level spectra for (**a**) Ca 2p and (**b**) P 2p from an N-doped TiO_2_ coating deposited on an 800 nm pore size membrane exposed to CaSO_4_·2H_2_O (120 mg/L Ca^2+^) and 1 mM phosphate buffer solution.

Without divalent cations (Mg^2+^ and Ca^2+^), the lower reaction rate constants at alkaline pH compared to neutral pH are presented in Section 2.1. At pH = 7, addition of Ca^2+^ resulted in ~30% decrease in reaction rate constant, while at pH 8.5, addition of divalent cations did not impact CBZ rate constants, compared to the control at the respective pH. Table 1 shows Ca^2+^ and Mg^2+^ concentrations measured before and after the photocatalytic degradation experiments, at pH 7 and 8.5. At pH 7 and 8.5, a decrease in ~20% Ca^2+^ concentration was observed after the experiments were completed; while at pH = 8.5, no significant change was detected in Mg^2+^ concentration. Calcium disappearance may indicate adsorption onto the negatively charged membrane surface due to electrostatic forces. Interestingly, accumulation of Ca^2+^ on the catalytic surface affected CBZ degradation only at pH 7 (adjusted by phosphate buffer). The steep decline in reaction rate constant in the presence of Ca^2+^ at neutral pH may be related to the presence of PBS. Hydrated phosphate ions are attracted to the hydrated TiO_2_ surface by hydrogen bridges and when adsorbed at the catalyst surface can serve as mediators for adsorption of Ca^2+^ [27,28,29]. To examine the presence of precipitation formed, the sample was exposed to a solution containing CaSO_4_·2H_2_O (120 mg/L Ca^2+^) and 1 mM phosphate buffer (pH = 7 adjusted using NaH_2_PO_4_/Na_2_HPO_4_) in DI water. The solution was recirculated through the membrane inside the filtration cell at a flow rate of 0.5 L/h, with continuous irradiation for 2 h. Afterwards, the sample was removed and packed in a glass container without rinsing with DI water or drying. X-ray photoelectron spectroscopy (XPS) analysis presented the following results, as shown in Figure 3: Calcium and phosphorous were found to be present with very similar concentrations, the Ca/P ratio being 1.06. The binding energy of the Ca 2p and P 2p peaks were 347.6 eV and 133.8 eV respectively. These peak positions correspond to calcium phosphate and show excellent agreement with the values given by Chusuei et al. [29]. The exact phase of calcium phosphate cannot be distinguished from the XPS core level spectra [29], but the Ca/P ratio of 1.06 would suggest di-calcium phosphate (CaHPO_4_ or CaHPO_4_·2H_2_O) rather than tri-calcium phosphate (Ca_3_(PO_4_)_2_). Therefore, a much denser layer of CaHPO_4_ or CaHPO_4_·2H_2_O precipitate as opposed to calcium sorption alone may be detrimental to photocatalytic active sites.

An additional experiment performed at pH 7 in the presence of Ca^2+^ but in the absence of phosphate buffer (i.e., pH adjusted by titration with NaOH/HCl) (data not shown), strengthened the hypothesis regarding the interaction between phosphate and Ca^2+^. No inhibiting effect on reaction rate was found at these conditions (without phosphate addition); however, the pH was not stable throughout the experiment (i.e., H^+^ generation by OH· radical production from water and radical attack on CBZ).

Magnesium and calcium are found naturally in water and are the main source of water hardness. These ions may inhibit pollutant degradation during photocatalysis [30,31]. Gupta et al. reported that Ca^2+^ (up to 200 mg/L) inhibited (~20%, at pH 6.4) the photocatalytic degradation of a mixture of methyl red (anionic) and crystal violet (cationic) dyes via Ag-doped TiO_2_ [32]. Furthermore, crystal violet, did not color the photocatalyst during photocatalysis. Thus, Ca^2+^ interferes with dye adsorption to the surface of the catalyst, in a way that inhibits the degradation process. Shirazi et al. found that Mg^2+^ and Ca^2+^ reduced CBZ degradation using TiO_2_/UV (at pH 7) by 7% and 20%, respectively [33]. Similarly Kashif and Ouyang, demonstrated a significant decrease in phenol degradation using TiO_2_/UV in the presence of Mg^2+^ and Ca^2+^ (at pH 5) [34]. The authors attributed this to the formation of phenol complexes that are difficult to degrade compared to phenol. Another study, however, showed that Mg^2+^ addition had negligible effects on glyphosphate degradation by TiO_2_ suspension [24].

In another study, Li et al. showed that the presence of Ca^2+^ or Mg^2+^ (at pH 7) greatly enhanced humic acid (HA) photocatalytic oxidation rate in a TiO_2_ suspension [35]. More than 2.5 h was needed to remove >90% of HA from solution without addition of Ca^2+^ or Mg^2+^, compared to less than 1 h with cation addition. At neutral pH, the catalyst and humic acid are both negatively charged, thus Mg^2+^ and Ca^2+^ ions may serve to neutralize the catalyst and the HA charges, reducing the repulsion between the two species. Additionally, Selvam et al. demonstrated that addition of Mg^2+^ (200 mg/L, pH = 4) enhanced 4-fluorophenol degradation by TiO_2_ suspension due to charge separation increase by Mg^2+^ conversion to Mg^+^, by accepting the electron from the conduction band [36]. To conclude, the current study showed that both the hardness cations in water (Ca^2+^ or Mg^2+^) and the pH are critical parameters to determine the CBZ degradation efficacy via PMR.

### 2.3. Effect of Dissolved Organic Matter (DOM)

The PCA of CBZ by N-doped TiO_2_-coated Al_2_O_3_ membrane was examined with the addition of DOM. Suwannee River Natural Organic Matter (SRNOM, 4 and 8 mg/L), Suwannee River Humic Acid (SRHA) (8 mg/L) and Suwannee River Fulvic Acid (SRFA) (8 mg/L), were separately added to buffered DI water spiked with 1 mg/L CBZ. The pH was adjusted to 7 by 1 mM PBS. The percentage decrease in reaction rate constant for each case is summarized in Table 2.

Increase in SRNOM concentration (4 to 8 mg/L) resulted in decrease in reaction rate constant (11% to 24%, respectively). Furthermore, a ~40% decrease in reaction rate constant was obtained with SRHA and SRFA. As the light screening effect of water absorbance in the presence of DOM was insignificant (<<1% decrease in incident irradiance for wavelength range between 256–500 nm), perhaps the detrimental effect of aromatic DOM, is due to competition of the various DOMs for OH· radicals.

In general, the presence of background DOM can enhance or inhibit photo-degradation, depending on the experimental conditions. Humic substances (HS) readily absorb light, inducing formation of reactive oxygen species as OH·, singlet oxygen, hydrogen peroxide, peroxy radicals, and solvated electrons. For example, Minero et al. studied the photochemical degradation of organophosphorus pesticides in the presence of HS that served as sensitizers or precursors for generation of photo-reactants. As a result, the photolysis of atrazine increased three fold under solar irradiation (with 10 ppm organic carbon) [37]. Okamura and Sugiyama demonstrated that natural HS from river waters and natural soils accelerated irgarol UVA photodegradation [38]. Cid et al. found an increase in TiO_2_/UV-mediated reduction of Cr (VI) to Cr (III) in the presence of HA, due to direct oxidation of HA by positive holes on the surface of TiO_2_, decreasing electron-hole recombination and increasing the electrons available for reduction of chromium [39].

On the other hand, organic matter can reduce the photodegradation rate. Selli et al. [40] showed a decrease in photocatalytic degradation (by 18% and 30%) of tetrachloroethene by TiO_2_ in the presence of HA (5 and 10 mg/L, respectively). The aromatic nature of humic acids (i.e., electron-rich molecules) is responsible for their tendency to act as photosensitizers. Upon excitation, photosensitizer at the semiconductor surface may inject an electron into the catalysts conduction band, initiating photocatalytic degradation [41]. Nienow et al. reported a greater inhibition effect in the presence of SRHA compared to SRFA on the degradation of lindane by UV/H_2_O_2_ [42]. Autin et al. found that the presence of organic compounds in the solution affected the OH· scavenging rate as a function of concentration and nature of the compounds. Therefore, an increase of the UV dose is required for metaldehyde degradation via UV/H_2_O_2_ or UV/TiO_2_ to overcome the scavenging effect [43].

### 2.4. Effect of Chloride

The PCA of CBZ by N-doped TiO_2_-coated Al_2_O_3_ membrane was examined at different chloride concentrations (100, 250, and 500 mg/L Cl^−^). Chloride was added from a stock solution of sodium chloride (NaCl) to a buffered DI water containing 1 mg/L CBZ. As shown in Table 3, negligible effect of increasing chloride concentrations on the degradation rate constant of CBZ was observed.

Although Cl^−^ addition to the treated solution did not result in either a positive or negative effect on CBZ photocatalytic oxidation, both effects were reported in the literature. Makita and Harata investigated the influence of Cl^−^ in seawater on photocatalytic decolorization of rhodamine B dye [44]. The maximum decolorization rate was found at moderate Cl^−^ concentration (2.0 wt %), presumably promoted by efficient production of Cl^−^ and OH· in addition to reduction in electron-hole recombination. Similarly, Yuan et al. found that Cl^−^ concentration between 0–200 mM enhanced the decolorization of azo dye, however further addition of Cl^−^ significantly decreased the reaction rate [45].

Chloride may react with the positive holes on the photocatalytic surface to produce chloride radicals (Equation (1)). The scavenging of OH· by Cl^−^, according to Equation (2), is initiated only at acidic water pH due to the transformation of ClOH·^−^ into Cl· which is reversible [46,47].

(1) $${\mathrm{Cl}}^{-}+h^{+}\to \mathrm{Cl}$$

(2) $${\mathrm{Cl}}^{-}+\mathrm{OH}\cdot ↔{\mathrm{ClOH}\cdot }^{-}+H^{+}↔\mathrm{Cl}\cdot +H_{2}O$$

A number of studies demonstrated the inhibiting effect of Cl^−^ on photocatalytic processes. Piscopo et al. investigated the influence of pH (5–6), and chloride ([Cl^−^] < 0.02 mol/L) on the degradation of benzamine by a suspension of TiO_2_ [48]. The predominant species at the experimental pH was suggested as Ti(OH_2_)^+^, thus Cl^−^ competing with benzamine for active sites on the catalyst decreased benzamine degradation. Similarly, Zhang et al., showed that competition of Cl^−^ for TiO_2_ active sites led to lower efficiency in photocatalytic reactions [17]. Okamoto et al. explained the inhibiting effect of Cl^−^ by its competition with oxygen for electrons, which reduces superoxide radicals formation therefore blocking the chain reaction for hydroxyl radical production [49].

### 2.5. Effect of Alkalinity

Alkalinity is defined as the capability of water to neutralize acids, and acts as a buffer to maintain a steady pH in natural water systems. Alkalinity is mostly comprised of bicarbonate (HCO_3_^−^), carbonate (CO_3_^2−^) and hydroxide (OH^−^). These ions may enter ground and surface water as the water flows over rocks, soils, and plants. Bicarbonate as well as carbonate anions deactivate OH· according to Equations (3) and (4) [50].

(3) $${\mathrm{HCO}}_{3}^{-}+\mathrm{HO}\cdot \to H_{2}O+CO_{3}^{-}\cdot$$

(4) $${\mathrm{CO}}_{3}^{2-}+\mathrm{HO}\cdot \to HO^{-}+CO_{3}^{-}\cdot$$

**Figure 4:**
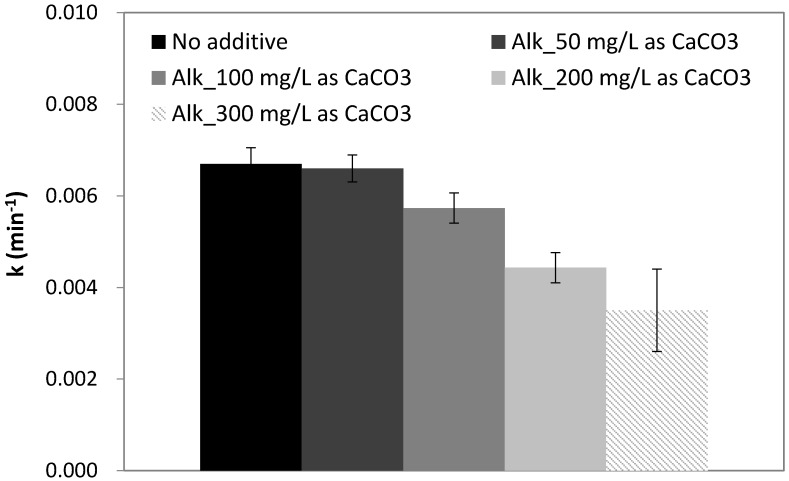
Degradation rate constant of CBZ (C_0_ = 1 mg/L) as a function of water alkalinity.

The effect of water alkalinity on PCA of CBZ by N-doped TiO_2_-coated membranes in the presence of different bicarbonate concentrations (50, 100, 200, and 300 mg/L as CaCO_3_) is presented in Figure 4. Stock solution of sodium bicarbonate (NaHCO_3_) was used to increase water alkalinity. The pH value was naturally sustained within 7 < pH < 8.5 by bicarbonate.

The degradation rate of CBZ was negatively affected only at moderate alkalinity concentrations of 100 mg/L as CaCO_3_ (122 mg/L HCO_3_^−^), with a decrease of 14% in reaction rate constant. At 200 mg/L as CaCO_3_ the reaction rate decreased drastically by 34%, while higher concentration of bicarbonate showed a minor additional decrease. Two simultaneous effects may occur: (1) bicarbonate scavenging effect on hydroxyl radicals and (2) increasing concentration of bicarbonate and consequently increasing carbonate radical anions which can contribute to CBZ degradation. Measurements of alkalinity concentration before and after the photocatalytic reaction showed a decrease of 15–25 mg/L as CaCO_3_ from initial concentration for all the concentrations examined, suggesting bicarbonate ions participate in the reaction.

As was shown in Section 2.1, an increase in pH value from neutral to 8.5 resulted in a decrease of ~25% in the CBZ degradation rate constant. As alkalinity is the cause of pH increase, the negative effect of bicarbonate may be partially ascribed to the change in pH rather than hydroxyl radical scavenging alone. Similarly, Pelaez et al., demonstrated that the addition of 50 mg/L Na_2_CO_3_ (pH = 10.3) inhibited microcystin-LR (MC-LR) degradation under visible light-activated TiO_2_ photocatalyst [51]. Phosphate buffer was used to reduce solution pH and allow differentiating between the negative contribution of alkaline pH and the presence of HCO_3_/CO_3_^2−^. No significant change in initial degradation rate of MC-LR was found at modified pH of 7.1. However, when increasing the Na_2_CO_3_ concentration to 150 mg/L in buffered solution, a reduction of 80% in the initial reaction rate was obtained compared to a concentration of 50 mg/L. Consequently, although carbonate and bicarbonate are scavengers of radicals, another phenomenon may occur by increasing the negative charge on the catalyst surface, when pH > PZC, thus impacting the degradation rate by inhibiting surface reactions. The equilibrium between bicarbonate and carbonate is pH dependent: the predominant ion at neutral water pH is bicarbonate, while at pH > 10 carbonate is the main ion [52]. Lair et al. [53] reported that carbonate inhibited both adsorption on TiO_2_ and the photocatalytic degradation rate of naphthalene. The inhibiting effect of both carbonate and bicarbonate ions was due to the scavenging of OH·. However, bicarbonate was found to scavenge 50-times less OH· than carbonate. Hence bicarbonate, which is common in natural waters (pH 6.5–8.5), is less inhibiting than other parameters (e.g., pH, NOM) at low concentrations.

In contrast, Hu et al. demonstrated that an increase in concentration of bicarbonate (over 500 mg/L NaHCO_3_, pH 9) led to an increase in TiO_2_-mediated degradation of sulfamethoxazole (SMX) [54]. The explanation relied on the significantly higher concentration of HCO_3_^−^ and CO_3_^2−^ compared to SMX; therefore, interacting more strongly with the catalyst and decreasing electron-hole recombination. In addition, as CO_3_^−^· are more stable with longer lifetime than OH·, these radicals are not limited to TiO_2_ surface reactions only and may diffuse and react in the bulk solution. However, carbonate radicals have a less positive oxidation potential compared to hydroxyl radicals and are highly selective to the drug target, and this may be significant for the differentiation between degradation kinetics of SMX compared to CBZ in photocatalysis.

### 2.6. Summary of Water Quality Results

The range of values tested for alkalinity, chlorides, pH levels, and organic matter represent the values typical of natural water as reviewed in the literature. The pH values of natural water (ground or surface water) typically vary between 6 to 8.5 [55], while alkalinity varies between 200–300 mg/L as CaCO_3_ [56]. NOM concentrations in surface and ground water are in the range of 2–8 mg C/L, depending on the type of soil or substrate [57]. The range of chlorides examined in this study were in accordance with the maximum contaminant level for chloride in drinking water [58]. A maximum concentration of 120 mg/L calcium was used corresponding to a total hardness of 300 mg/L as CaCO_3_. The upper range of hardness values was chosen to correspond to the total hardness value found for Lake Kinneret (Sea of Galilee, Israel) [59]. Magnesium (Mg^2+^), present also in natural water and especially significant in groundwater, was examined at an equivalent concentration to Ca^2+^ for comparison between the divalent cations.

A summary of all the water quality parameters examined and their impact on the photocatalytic degradation reaction rate, is presented in Table 4.

A previous study showed six main by-product compounds identified following photocatalytic degradation for CBZ using N-doped TiO_2_ after prolonged irradiation [60]. The identified compounds revealed two pathways to CBZ degradation: hydroxylation of CBZ at the 10 position following formation of 10,11-epoxycarbamazepine (V) or OH· attack on the aromatic ring leading to hydroxylated derivatives (III, IV) (i.e., OH· addition) [61,62]. However, these pathways were identified at extended irradiation times, and pathways for CBZ degradation could differ at short irradiation times and for different water chemistries, as in the current study.

### 2.7. Membrane Regeneration by Chemical Cleaning

Photocatalytic membranes may be subjected to a reduction in PCA due to saturation of active sites by organic and inorganic substances in water. Although photocatalytic surfaces have a potential self-cleaning ability, removing organics adsorbed to the catalyst by exposing the coated surface to prolonged irradiation can be impractical. Moreover, self-cleaning will not be effective for overcoming the inorganic salt precipitation on the catalytic surface.

**Figure 5:**
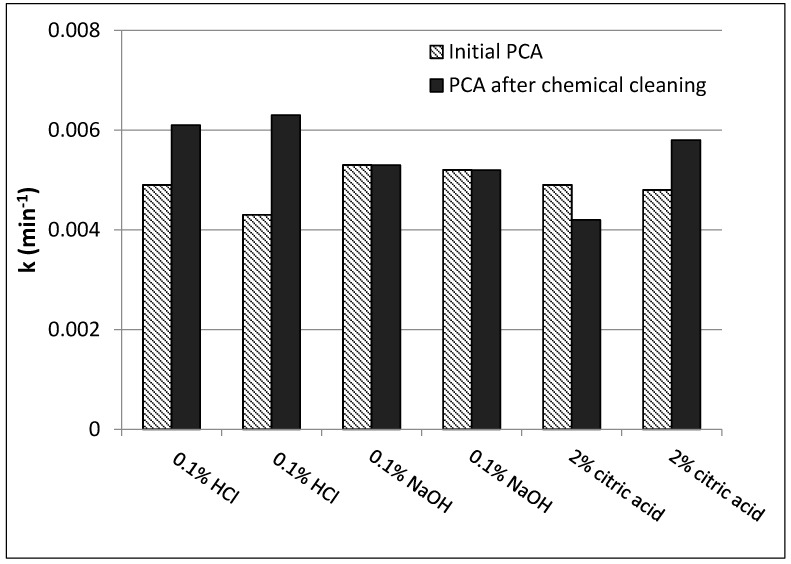
Initial reaction rate constant versus rate constant after 2 h immersion in: 0.1% HCl, 0.1% NaOH or 2% citric acid.

Regeneration in activity of N-doped TiO_2_-coated Al_2_O_3_ membranes was examined by subjecting the membranes to chemical cleaning after continuous use. Three typical cleaning agents were examined: citric acid (2%), HCl (0.1%) as an inorganic foulant cleaning agents and NaOH (0.1%) as an organic foulant cleaning agent. To determine whether the cleaning agents chosen had a detrimental effect on photocatalytic coating, newly coated photocatalytic membranes were tested for initial PCA. Afterwards, each membrane was soaked in a different cleaning solution for 2 h, following flushing with DI water to remove cleaning agent residuals and finally tested for PCA. From the results presented in Figure 5 no detrimental effect on PCA was observed for 0.1% *w*/*w* HCl and 0.1% *w*/*w* NaOH. However, an inconsistent trend was observed for membranes cleaned by 2% *w*/*w* citric acid with a reduction in PCA in some cases that can perhaps be attributed to the organic nature of the acid, as citric acid can adsorb to the photocatalytic surface and subsequently reduce PCA. Membranes cleaned by 0.1% *w*/*w* HCl showed a significant increase in PCA with up to 30% increase in reaction rate constant. This increase can be due to removal of contaminants either from the deposition procedure or from the membrane manufacturing process. Membranes treated with 0.1% *w*/*w* NaOH showed no change in their initial PCA.

**Figure 6:**
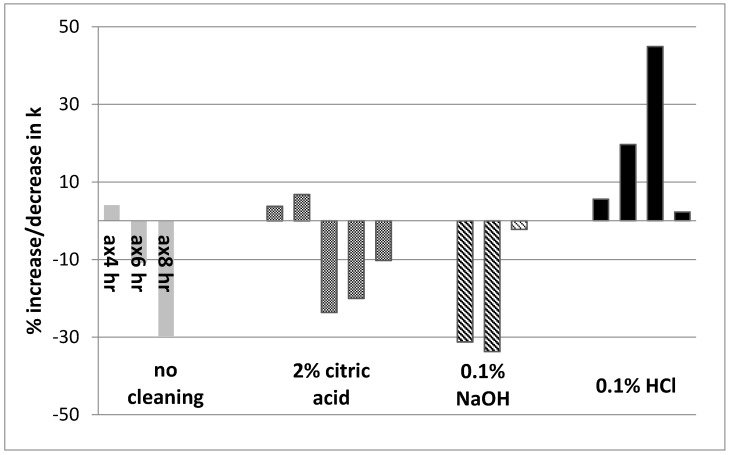
Percentage increase/decrease in k for uncleaned membrane and membranes cleaned by 2% (*w*/*w*) citric acid, 0.1% (*w*/*w*) NaOH and 0.1% (*w*/*w*) HCl after 8 h of continuous use.

Regeneration of the N-doped TiO_2_-coated membranes was evaluated after 8 h of continuous use by immersing the used membranes in the cleaning agents following thorough rinsing with DI water and finally a PCA test. Figure 6 presents the percentage increase or decrease in reaction rate constant, k, for uncleaned and cleaned used membranes. The uncleaned membrane showed a gradual decrease in PCA with continuous use (up to 30% after 8 h continuous operation). The cleaned membranes represent individual membranes cleaned after 8 h operation. Out of the five membranes cleaned by citric acid three showed a decrease of 10–24% in PCA, confirming the results on new (unused) membranes. Membranes cleaned by 0.1% NaOH did not regenerate and remained at ~30% lower PCA with the exception of one membrane. All membranes were successfully regenerated by 0.1% HCl with an increase in PCA ranging between 5–45%. Although sodium hydroxide did not show regeneration of PCA in laboratory controlled experiments, it can still prove efficient in organic fouling removal under real conditions.

Little literature can be found on catalyst regeneration and reuse by chemical cleaning. Reduction in PCA with long-term use was reported by Rao et al. and attributed to adsorption of reactants and by products to the catalyst. Chemical cleaning by hypochlorite after biological fouling successfully regenerated the immobilized TiO_2_ to its initial efficiency [63]. Nakano et al. showed a decrease in porous TiO_2_/SiO_2_ catalyst activity gradually with time, when used for dinitrophenol degradation in a fixed bed reactor under solar light [64]. They attributed the decrease in activity to fouling by adhesion of metals to the catalytic surface due to the change in the catalyst color from white to brown. The regeneration of the PCA was examined by inserting the fouled catalyst in citric acid, hydrochloric acid, nitric acid, and deionized water for one week. The catalytic activity after regeneration showed no recovery of the catalyst for samples dipped in DI water. However, all acids showed a significant PCA recovery while hydrochloric acid cleaning was found the most efficient with ~90% recovery. Miranda-García et al. evaluated four different strategies for regeneration of TiO_2_-coated glass spheres, used for photocatalytic degradation of 15 organic contaminants [65]. Fouling of the catalyst was observed after consecutive treatment cycles, requiring longer reaction time. The regeneration treatments employed were H_2_O_2_/UV, NaOH, NH_4_OH, and calcination (at 400 °C). Effective regeneration of PCA was achieved after treating with H_2_O_2_/UV or calcination while NaOH was found to partially remove the catalyst layer.

The Al_2_O_3_ membranes purchased from the manufacturer did not fit the experimental flow-cell dimensions, thus they were pre-cut in a local workshop prior to applying the photocatalytic coating; therefore, exposing the periphery. Membrane integrity after cleaning was determined by ICP-OES analysis. Membranes were first immersed in DI water followed by soaking in 0.1% *w*/*w* HCl solution for 2 and 12 h. The acidic environment after 2 and 12 h resulted in dissolution of ~3 mg/L Al and ~7 mg/L Al, respectively. These results lead to the conclusion that future research on cleaning conditions is required. After confirming the photocatalytic membrane’s regeneration by HCl, the optimization should consider three aspects: cleaning duration (shorter exposure time), acid concentration (reducing concentration), acid type and pre-cutting membranes by the manufacturer rather than at a local workshop.

### 2.8. Surface Analysis

**Figure 7:**
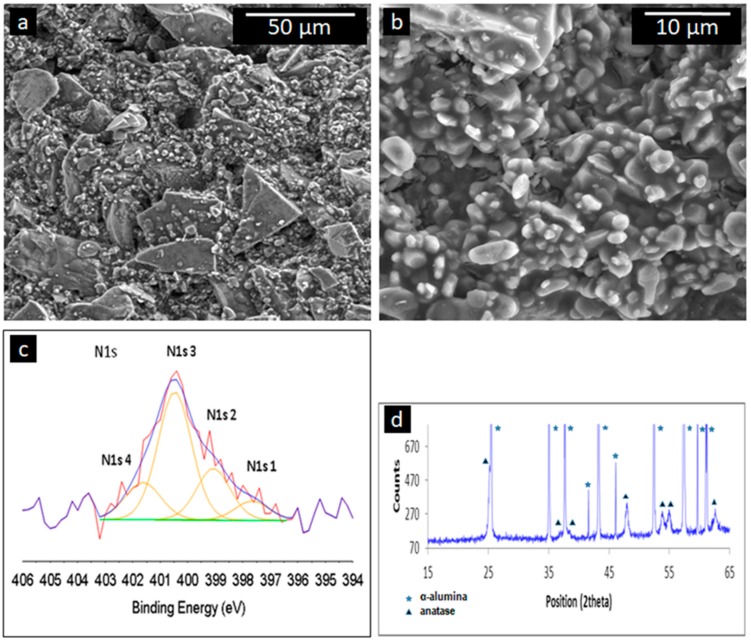
Material characterization results of the N-doped TiO_2_ coated membrane: (**a**,**b**) scanning electron microscopy (SEM) images; (**c**) a peak fitted XPS N 1s spectrum; (**d**) glancing-angle X-ray diffraction (GAXRD) diffractogram showing the presence of the nanocrystalline anatase based coating and the underlying α-alumina membrane.

Scanning electron microscopy (SEM) micrographs of the N-doped TiO_2_ coated 800 nm pore size alumina membrane are shown in Figure 7. A lower magnification micrograph is given in Figure 7a and a higher magnification image shown in Figure 7b. It can be seen from the SEM images, that the membrane is comprised of large particles, decorated with smaller particulates, typically in the range of 1 to 5 µm in diameter. The N-doped TiO_2_ coating did not exhibit a morphology that enabled it to be clearly identified from the SEM images. However, the GAXRD diffractogram given in Figure 7d, clearly shows the presence of the nanocrystalline anatase based coating on the α-alumina membrane. The average grain size of the coating was calculated to be 17 nm (from the (200) reflection at 2θ = 48.05°).

XPS spectra were recorded from several coated membranes, deposited with nominally the same sol-gel process. The nitrogen concentration in the N-doped TiO_2_ coating varied between 0.2 and 0.4 at%. A typical peak fitted N 1s spectrum is shown in Figure 7c. The spectrum was fitted with four components, labelled N 1s1 to N 1s4. The low intensity of the N 1s peak limits the reliability of the peak fit. Nevertheless, some general comments can be made. The strongest component, N 1s3 at around 400 eV component occurs on many surfaces due to the presence of N-containing organic contamination [66] but a peak at a similar binding energy was attributed to interstitial NO^−^ species in the bulk [67].The N 1s4 component with a binding energy between 401.5–402.0 eV is associated with surface contamination [66]. The two lower energy peaks, N 1s1 and N 1s2, occur at binding energies between 397.5 and 399.0 eV. Peaks with such binding energies are generally ascribed to the presence of interstitial N species [68,69,70]. There is no N peak at a binding energy at around 396 eV, attributable to substitutional N [66]. Hence, in these N-doped TiO_2_ coatings, the N is present as interstitial rather than substitutional species.

## 3. Materials and Methods

### 3.1. Carbamazepine as a Model Pollutant

Carbamazepine, an antiepileptic drug, is one of the most frequently detected compounds in the aquatic environment owing to its resistance to biodegradation [62,71]. Carbamazepine is hydrophobic, a neutrally charged drug, relatively polar and does not adsorb readily to soils and sediments [72]. The persistence of CBZ in the environment led to its use as a marker contaminant in groundwater [73]. The physical and chemical data of CBZ are shown in Table 5:

### 3.2. Chemicals and Reagents

A stock solution of carbamazepine (>99% purity, Sigma-Aldrich, St. Louis, MO, USA) was prepared by dissolving in deionized (DI) water (Direct-Q3 UV system, Millipore-France, Molsheim, France) at a concentration of 50 mg/L. Stock solutions of A—0.1 M sodium phosphate monobasic and B—0.1 M sodium phosphate dibasic from Sigma Aldrich (Darmstadt, Germany) were used for the preparation of a pH 6 (A:B = 87.12:12.3 respectively, volumetric ratio) and 7 (A:B = 39:61 respectively, volumetric ratio) buffers at concentration of 1 mM at room temperature. Stock solutions of 225 mL boric acid (50 mM)—Stock A and 7.5 mL sodium tetra-borate (50 mM)—Stock B from Sigma Aldrich (Darmstadt, Germany) were used to form 2 mM borate buffer at pH = 8.5.

Alkalinity of the water was achieved by using NaHCO_3_ (Sigma-Aldrich, Darmstadt, Germany). Chloride was added by dissolved NaCl (Sigma-Aldrich, Darmstadt, Germany). Calcium was added by dissolved CaSO_4_·2H_2_O (Sigma-Aldrich, Darmstadt, Germany). Magnesium was added by dissolved MgSO_4_ (Sigma-Aldrich, St. Louis, MN, USA). Suwannee River natural organic matter (SRNOM), Suwannee River Humic Acid (SRHA) and Suwannee River Fulvic Acid (SRFA) (International Humic Substances Society-IHSS, St. Paul, MN, USA) were used, individually, to simulate dissolved organic matter. The listed above DOMs were dissolved in DI water to a concentration of 500 mg/L at pH 10 (adjusted by NaOH) following filtration through a 0.45 μm cellulose acetate filter, and used as stock solutions for the experiments.

Membrane regeneration experiments were conducted using citric acid (Gadot Biochemical Industries Ltd., Haifa Bay, Israel), hydrochloric acid 32% (Bio Lab Ltd., Jerusalem, Israel) and sodium hydroxide (Merck KGaA, Darmstadt, Germany, analytical grade).

### 3.3. Deposition Technique

N-doped TiO_2_ coating was deposited by the sol-gel method as previously described [12], onto a commercial flat α-Al_2_O_3_ MF membrane with a nominal pore size of 800 nm (Nanostone Water, formerly KSM Water GmbH, Halberstadt, Germany). The effective filtration area of the membrane is 2.2 × 4.2 cm^2^, and the membrane thickness is 6.6 mm. In brief, the sol solution composed of isopropanol, tetrabutyl orthotitanate, triethanolamine, and ammonium hydroxide was applied by a drop-coating (0.12 mL cm^−2^ volume to membrane area), followed by annealing at 450 °C for 1 h.

### 3.4. Photocatalytic Activity of N-Doped TiO_2_-Coated Al_2_O_3_ Membrane

The PCA experiments were conducted using a custom-made filtration cell, enclosing the coated membrane. A detailed description and schematic drawing of the system set-up can be found in our previous work [12,77]. In brief, 200 mL of feed solution containing CBZ (DI water, 1 mg/L, with/without additives) was recirculated through the pressurized filtration cell for 30 min for equilibration, without irradiation. Afterwards, the feed solution was recirculated through, at a flow rate of 0.5 L/h, with continuous solar simulated irradiation for 2 h. The system was operated in a dead-end filtration mode. A 300 W ozone-free Xe lamp solar simulator (Newport, RI, USA) was used to achieve a consistent irradiation intensity of ~770 W/m^2^ (integrated between 280–700 nm wavelengths) throughout the experiments. Carbamazepine was detected and quantified by High Performance Liquid Chromatography following a method reported by Horovitz et al. [12]. The degradation rate of CBZ was fitted with the Langmuir-Hinshelwood (L-H) kinetics model [13,72], which was simplified to an apparent first order equation due to the low pollutant concentration (1 mg/L) as follows:

(5) $$k=ln(\frac{C_{t}}{C_{o}})/t$$

where *C*_0_ is the initial CBZ concentration after equilibration in the dark and *C_t_* is the concentration after *t* minutes of irradiation (mg/L). *k* is the apparent reaction rate constant (min^−1^) for CBZ.

### 3.5. Water Analysis

Concentrations of chloride, alkalinity, and total hardness were measured by titration (HACH digital titrator, model 16,900, Loveland, CO, USA), methods 8206, 8203, and 8213, respectively. The dissolved organic carbon (DOC) fraction was determined according to a method reported by Avisar et al. [2]. The obtained DOC level was ~50% of the SRNOM concentration used.

### 3.6. Surface Characterization

The SEM micrographs were acquired with a Quanta FEG 200 microscope by FEI, operating in a high vacuum environment with a beam voltage of 20 keV. Glancing angle X-ray diffraction (GAXRD) data was recorded using a Panalytical X’Pert diffractometer employing a glancing angle attachment at an incident angle of 1°. The X-ray source was Cu Kα radiation (λ = 1.5406 Å, 40 mA, 45 kV) and the diffractograms were recorded over a 2Θ range of 10–65°. The average grain size was calculated using the Scherrer equation:

(6) D=kλβcosθ

where *D* is the average grain size, *k* is the shape factor (a constant, given a value of 0.9), *λ* is the X-ray wavelength, *β* is the full width half maximum of the XRD peak and *θ* is the peak Bragg angle. It was assumed that peak broadening was due to grain size effects only.

XPS analysis was carried out using a Thetaprobe instrument (Thermo Fisher, East Grinstead, UK) employing Al Kα X-rays (hν = 1486.6 eV) at a power of 140 W and an X-ray spot size of 400 µm. The electron analyser was operated in a constant energy mode at a pass energy of 50 eV for the high resolution elemental spectra. Charge correction was based on adventitious carbon at 285.0 eV. Curve fitting was undertaken using the manufacturer’s Avantage software v4.74, after subtraction of a Shirley background and quantification was performed using instrument modified Wagner sensitivity factors.

## 4. Conclusions

N-doped TiO_2_-coated Al_2_O_3_ membrane showed a high potential to remove carbamazepine, a persistent pharmaceutical compound, from deionized water, under simulated solar irradiation. Investigating the effect of different water constituents on the photocatalytic oxidation provided important information regarding the potential efficiency of the process when treating water with different characteristics. The results indicate that the highest degradation rate of CBZ was observed at a neutral pH. When the pH level became acidic or basic the reaction rate decreased. Addition of Ca^2+^ and Mg^2+^ at alkaline conditions had no significant effect on CBZ degradation. However, presence of Ca^2+^ at neutral pH adjusted by phosphate buffer resulted in ~30% decrease in photocatalytic degradation. This result suggests that calcium hardness in combination with phosphate ions in the water resulted in deposition of CaHPO_4_ or CaHPO_4_·2H_2_O CaPO_4_ precipitate on the catalyst surface, thus screening the active sites. In the presence of three different DOMs tested, CBZ photocatalytic degradation was inhibited. Bicarbonate ions inhibit photocatalytic oxidation by N-doped TiO_2_-coated Al_2_O_3_ by two different routes: (i) low alkalinity concentration—bicarbonate-mediated water pH increase, (ii) high alkalinity concentration—hydroxyl radical scavenging by the bicarbonate to produce carbonate radicals. The presence of Cl^−^ ions had no effect on CBZ degradation. The N-doped TiO_2_ coated onto an Al_2_O_3_ membrane showed durability in PCA when exposed to chemical cleaning agents (citric acid, HCl, and NaOH). The regeneration of membrane PCA after continuous use was achieved by using HCl. Nevertheless, dissolution of metals such as Al and Ti should be monitored following acid cleaning. Membrane integrity after exposure to the cleaning agents should be thoroughly investigated before use in the field.

## Figures and Tables

**Table 1:** Concentrations of Ca^2+^ and Mg^2+^ during the photocatalytic experiment.

Phase of Experiment	Ca^2+^ 120 mg/L		Mg^2+^ 120 mg/L
	pH = 7	pH = 8.5	pH = 8.5
Initial	118.8 ± 4	118.8 ± 4	121 ± 2
Final	97.1 ± 0.7	96.4 ± 1	117.8 ± 0.5

**Table 2:** Percent decrease in carbamazepine (CBZ) degradation rate constant (k) in the presence of Suwannee River Natural Organic Matter (SRNOM) (4 and 8 mg/L), Suwannee River Humic Acid (SRHA) (8 mg/L) and Suwannee River Fulvic Acid (SRFA) (8 mg/L).

Rate Constant Change with DOM	4 mg/L SRNOM	8 mg/L SRNOM	8 mg/L SRHA	8 mg/L SRFA
% decrease in k	11	24	40	37

**Table 3:** CBZ degradation rate constants at different Cl^−^ concentrations.

[Cl^−^]	No Additive	100 mg/L	250 mg/L	500 mg/L
k (min^−1^)	0.0082 ± 0.00019	0.0088 ± 0.0006	0.0082 ± 0.00005	0.0086 ± 0.0011

**Table 4:** Summary of water type and composition effect on photocatalytic degradation reaction rate.

Water Type	pH	Alkalinity (mg/L as CaCO_3_)	Hardness (mg/L as CaCO_3_)	Ca^2+^ (mg/L)	Mg^2+^ (mg/L)	Chlorides (mg/L Cl^−^)	DOC (mg/L)	% Increase/Decrease in k
Lake Kinneret	8.5	84	305	48	41	310	4.2	−44
DI + Magnesium	8.5	-	-	-	120	-	-	−2.3
DI + Calcium	8.5	-	300	120	-	-	-	-
DI + Calcium	7	-	300	120	-	-	-	30.5
DI + Chloride	7	-	-	-	-	100	-	8.4
	7	-	-	-	-	250	-	1.6
	7	-	-	-	-	500	-	7.2
DI + Alkalinity	7	50	-	-	-	-	-	−1.5
	7	100	-	-	-	-	-	−14.4
	7	200	-	-	-	-	-	−33.8
	7	300	-	-	-	-	-	−48
DI + SRNOM	7	-	-	-	-	-	4	−11
	7	-	-	-	-	-	8	−24
DI + SRFA	7	-	-	-	-	-	8	−37
DI + SRHA	7	-	-	-	-	-	8	−40

**Table 5:** Main physicochemical properties of CBZ.

Structure and Formula	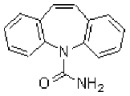 C_15_H_12_N_2_O	References
Molecular weight	236.3 g/mol	[74]
LogK (octanol-water)	2.45	[74,75]
pK_a_	13.9	[25,75]
Elimination half-life	25–65 h	[74,76]
